# Correction: Controlled intra- and extracellular localization of bioorthogonal polymeric nanozymes

**DOI:** 10.1039/d6sc90100j

**Published:** 2026-05-05

**Authors:** Cristina-Maria Hirschbiegel, Mathangi Shrikanth, Yagiz Anil Cicek, Nourina Nasim, Joe Truong, Junwhee Yang, Alexander Ribbe, Maged Abdelaziz, Vincent M. Rotello

**Affiliations:** a Department of Chemistry, University of Massachusetts Amherst 710N. Pleasant St. Amherst MA 01003 USA rotello@umass.edu; b Department of Polymer Science and Engineering, University of Massachusetts 120 Governors Drive Amherst MA 01003 USA

## Abstract

Correction for ‘Controlled intra- and extracellular localization of bioorthogonal polymeric nanozymes’ by Cristina-Maria Hirschbiegel *et al.*, *Chem. Sci.*, 2026, **17**, 4050–4060, https://doi.org/10.1039/D5SC07223A.

The authors regret that the units reported in Fig. 2c and 5 were mislabelled. The correct units are nmol nmol^−1^ instead of nmol µmol^−1^ for Fig. 2c and pmol and pmol h^−1^ instead of ng and ng h^−1^ for Fig. 5. The updated figures are shown herein. These errors do not affect the underlying data, analysis, results, and conclusions of the paper.

**Fig. 2:**
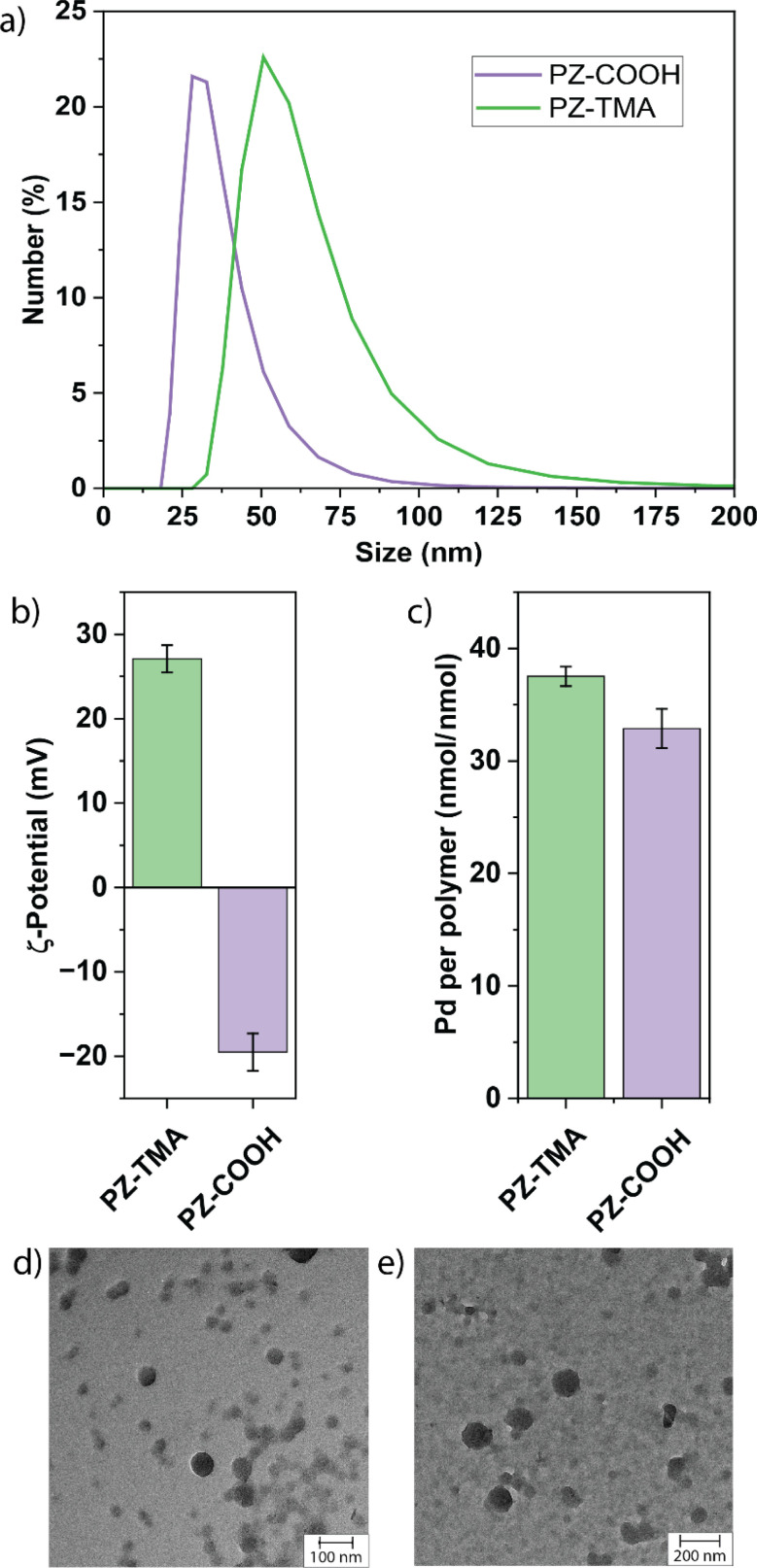
(a) Size distribution measured by dynamic light scattering (DLS); (b) *ζ*-potential of respective PZs; (c) quantification of encapsulated Pd^106^ measured by inductively coupled plasma mass spectrometry (ICP); TEM images of (d) PZ-TMA and (e) PZ-COOH.

**Fig. 5:**
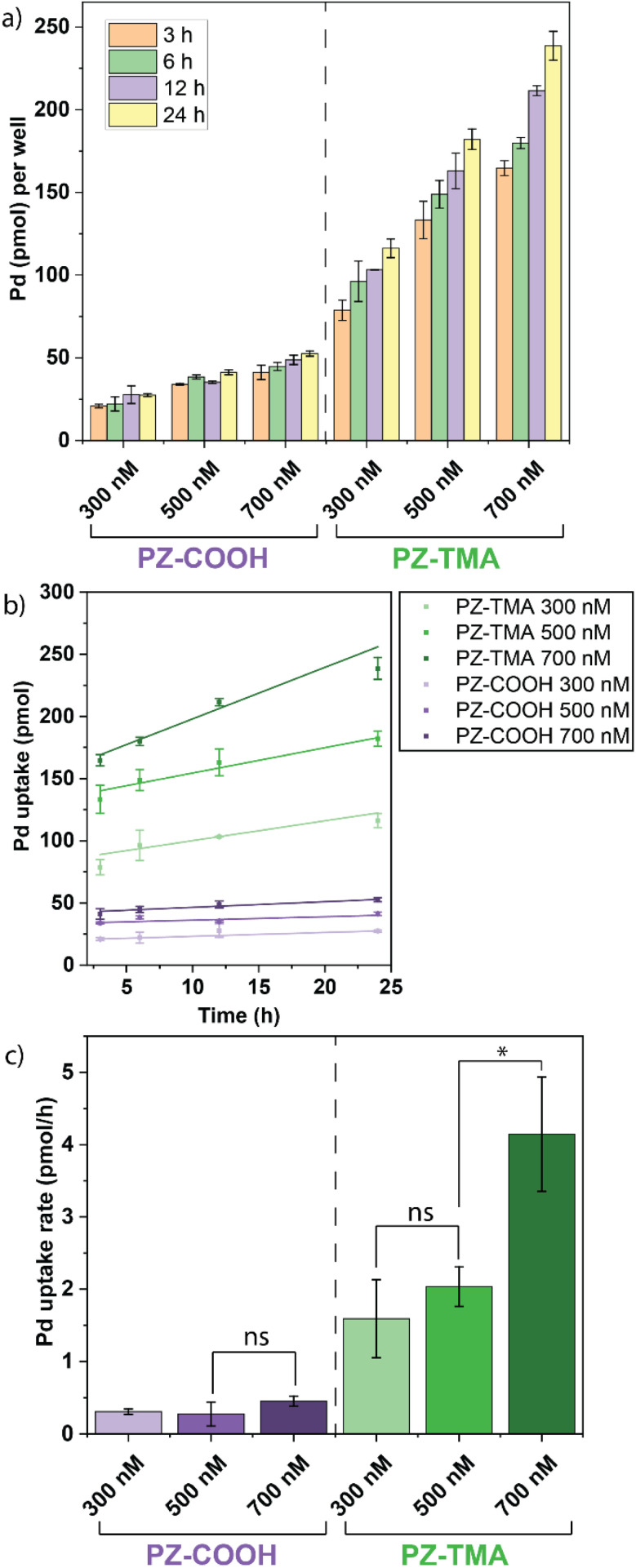
(a) Quantification of Pd^106^ in HeLa cells by ICP-MS at different time points of incubation with respective PZ; (b) linear fit of time-dependent uptake within cells; (c) average uptake of Pd (pmol h^−1^) in HeLa cells per PZ at different concentrations, demonstrating significantly higher uptake rates of positively charged PZ-TMA. All values represent the average of three individual measurements; error bars represent the standard deviation. Statistical analysis was performed using Student's *t*-test. ns = not significant; * = *p* < 0.05.

The Royal Society of Chemistry apologises for these errors and any consequent inconvenience to authors and readers.

